# Highly Sensitive and Selective Sensing of H_2_S Gas Using Precipitation and Impregnation-Made CuO/SnO_2_ Thick Films

**DOI:** 10.1186/s11671-021-03530-1

**Published:** 2021-04-28

**Authors:** Pimpan Leangtanom, Anurat Wisitsoraat, Kata Jaruwongrangsee, Narong Chanlek, Adisorn Tuantranont, Sukon Phanichphant, Viruntachar Kruefu

**Affiliations:** 1 Applied Chemistry Program, Faculty of Science, Maejo University, Chiang Mai, 50290 Thailand; 2 Nanoscience and Nanotechnology Program, Faculty of Science, Maejo University, Chiang Mai, 50290 Thailand; 3 Applied Physics Program, Faculty of Science, Maejo University, Chiang Mai, 50290 Thailand; 4 National Security and Dual-Use Technology Center, National Science and Technology Development Agency, Klong Luang, Phathumthani, 12120 Thailand; 5 Opto-Electrochemical Sensing Research Team (OEC), National Electronics and Computer Technology Center (NECTEC), Pathumthani, 12120 Thailand; 6 Synchrotron Light Research Institute, Nakhon Ratchasima, 30000 Thailand; 7 Center of Advanced Materials for Printed Electronics and Sensors, Materials Science Research Center, Faculty of Science, Chiang Mai University, Chiang Mai, 50200 Thailand

**Keywords:** Precipitation/impregnation, CuO/SnO_2_ thick films, Gas sensor, Sensing mechanism, Hydrogen sulfide

## Abstract

In this work, CuO-loaded tetragonal SnO_2_ nanoparticles (CuO/SnO_2_ NPs) were synthesized using precipitation/impregnation methods with varying Cu contents of 0–25 wt% and characterized for H_2_S detection. The material phase, morphology, chemical composition, and specific surface area of NPs were evaluated using X-ray diffraction, transmission electron microscopy, scanning electron microscopy, energy-dispersive X-ray spectroscopy, X-ray photoelectron spectroscopy, and Brunauer–Emmett–Teller analysis. From gas-sensing data, the H_2_S responses of SnO_2_ NPs were greatly enhanced by CuO loading particularly at the optimal Cu content of 20 wt%. The 20 wt% CuO/SnO_2_ sensor showed an excellent response of 1.36 × 10^5^ toward 10 ppm H_2_S and high H_2_S selectivity against H_2_, SO_2_, CH_4_, and C_2_H_2_ at a low optimum working temperature of 200 °C. In addition, the sensor provided fast response and a low detection limit of less than 0.15 ppm. The CuO–SnO_2_ sensor could therefore be a potential candidate for H_2_S detection in environmental applications.

## Background

Hydrogen sulfide (H_2_S) is a highly toxic gas widely produced from several sources, such as manure fermentation plants, wastewater treatment systems, petroleum refineries, landfill sites, textile factories, stagnant water wells, extruded rubber industries, and other similar industrial facilities. H_2_S can be adsorbed through human lungs, gastrointestinal regions, and normal skin. Its funky odor will freeze the sense of smell leading to immediate paralysis and mortality when its concentration exceeds its threshold limit value (TLV) of 10 ppm [1]. Therefore, it is compelling to develop an effective and low-cost gas sensor that can detect H_2_S at sub-ppm concentrations with high response, short response time, high selectivity and good stability.

Semiconducting metal oxides, such as zinc oxide (ZnO), tin dioxide (SnO_2_), titanium dioxide (TiO_2_) and nickel oxide (NiO), have been extensively studied for various gas-sensing applications [2–6]. Among them, tin dioxide (SnO_2_) has been regarded as the most promising n-type metal oxide gas-sensing material due to its low cost, diverse gas response, ease of doping, high chemical stability, and wide range of working temperature from 100 to 600 °C [7–9]. In particular, it has been reported as one of the most attractive candidates for H_2_S detection after the modification by doping with metallic dopants [10–18], loading with metal oxide nanoparticles [19–27], and forming nanocomposites with different metal oxide semiconductors [28, 29]. However, the H_2_S-sensing performances of SnO_2_ still need further improvements.

Copper Oxide (CuO) is a functional p-type metal oxide semiconductor with a moderate energy gap of 1.2–2.0 eV, remarkable sensitivity and selectivity toward H_2_S. CuO-loaded SnO_2_ gas sensors have been extensively characterized toward H_2_S as presented in Table 1. CuO dopants provides relatively high enhancement of H_2_S response and selectivity for SnO_2_ sensors [19–27]. The H_2_S-sensing performances also depend substantially on the synthesis method and the form of metal oxide materials. From the table, recent reports CuO/SnO_2_ sensors are mostly in thick-film and thin-film forms, which offer similarly competitive H_2_S-sensing performances depending on synthesis method and preparation parameters. Between them, thick-film sensors are more preferred in practical applications due to their much lower production cost. Hence, it is compelling to investigate the H_2_S-sensing properties of thick-film CuO/SnO_2_ materials prepared by other advanced techniques.

**Table 1:** A summary of H_2_S response of metal-loaded SnO_2_ and CuO/SnO_2_ nanomaterials prepared by various methods

Materials	Form	Technical used	Gas conc. (ppm)/Temp (°C)	H_2_S Response	Refs
3.0 mol% Ag–SnO_2_	Thick film	Spray pyrolysis	450/100	1.38	[10]
0.1 wt% V–SnO_2_	Thick film	Flame spray pyrolysis and spin coating	10/350	2.27 × 10^3^	[11]
0.5 wt% Mo–SnO_2_	Thick film	Flame spray pyrolysis and spin coating	10/250	≈105	[12]
Sb–SnO_2_ nanoribbons	Thin film	Thermal evaporation	100/150	≈55	[13]
0.64 at% Fe–SnO_2_	Thin film	Rheotaxial grown and Thermal oxidation	10/225	14.5	[14]
Cu-doped SnO_2_	Thick film	Ultrasonic spray pyrolysis	95.9/100	7.24 × 10^3^	[15]
2mol% Cu–SnO_2_	Thick film	Hydrothermal and drip coating	300/300	40	[16]
Cu–SnO_2_ nanowires	Thin film	Thermal evaporation	10/150	5 × 10^5^	[17]
1 at% Cu–SnO_2_	Thick film	Electrostatic sprayed	10/100	2.5 × 10^3^	[18]
SnO_2/_CuO islands	Thin film	Sputtering	5/250	128	[19]
CuO-loaded SnO_2_	Thick film	Ultrasonic spray pyrolysis	1/300	22.4	[20]
CuO/SnO_2_	Thin film	Chemical vapour deposition	10/250	26.3	[21]
3 vol% CuO–SnO_2_	Thin film	Pulsed laser deposition	20/140	2.7 × 10^4^	[22]
CuO-loaded SnO_2_	Thin film	Electrospinning	10/300	1.98 × 10^4^	[23]
5 mol% CuO/SnO_2_	Thin film	Co-dissolution and electrospinning	1/200	≈23	[24]
SnO_2_–CuO	Thin film	Sputtering	20/150	8 × 10^3^	[25]
CuO–SnO_2_	Thin film	Pulsed laser deposition	20/100	2.3 × 10^3^	[26]
CuO–SnO_2_ nanowire	Thick film	Thermal evaporation	20/300	809	[27]
CuO–SnO_2_	Thick film	Precipitation/Impregnation and drop coating	50/200	6.7	[28]
20 wt% CuO/SnO_2_	Thick film	Precipitation/Impregnation and spin coating	10/200 10/150	1.359 × 10^5^ 3.1 × 10^4^	This work

Precipitation and impregnation are attractive methods for production of thick-film nanocomposite materials because of ability to form diverse nanostructures, low processing temperature, and low cost. Some CuO-loaded SnO_2_ nanomaterials synthesized by precipitation with NH_3_ precipitant and impregnation were studied for H_2_S gas-sensing. However, the reported results still offered only modest response at high H_2_S concentrations due possibly to large particle sizes [28]. Herein, precipitated SnO_2_ nanoparticles (NPs) were prepared using NaOH as a precipitant and impregnated with CuO at over a wide range of Cu contents to attain small nanoparticles and large responses at relatively low H_2_S concentrations. Thick-film sensors were fabricated by spin coating powder paste of synthesized CuO/SnO_2_ nanoparticles and the effects of CuO loading level on H_2_S-sensing properties were explained based on CuO/SnO_2_ heterojunctions.

## Methods

### Synthesis and Characterization of Nanoparticles

All chemicals with analytical grade were used directly without additional purification. Tin (IV) chloride pentahydrate (SnCl_4_·5H_2_O) as a tin source was dissolved in deionized (DI) water under constant stirring to obtain the 0.1 M aqueous solution. An appropriate volume of 0.1 M sodium hydroxide (NaOH) aqueous solution was slowly dropped onto the SnCl_4_·solution under vigorous stirring until white slurry appeared at the pH of 11. The slurry was washed thoroughly with DI water several times under centrifugation to remove chloride residues from the precipitate. The resulting precipitate was subsequently dried at 80 °C for 10 h in an oven and the obtained particles were calcined for 2 h at 600 °C at a heating rate of 10 °C/min. To impregnate CuO onto SnO_2_ nanoparticles, 0.872 g of copper (II) acetate hydrate (98%; Aldrich) was dissolved in 30 mL of ethanol under vigorous agitation. The solution was then dropped onto 0.5 g of SnO_2_ NPs with varying Cu concentrations from 5 to 25 wt%. Next, the suspension was continuously stirred until turning into homogeneous slurry and baked at 60 °C for 2 h in an oven. The resulting powders were annealed for 4 h at 300 °C at a heating rate of 10 °C/min.

The structural characteristics of NPs were evaluated using X-ray diffraction (XRD) with a Cu *Kα* (*λ* = 1.54056 Å) X-ray source. The surface morphology and elemental distributions of NPs were examined using high-resolution scanning transmission electron microscopes (HR-TEM). The oxidation states of materials were studied using X-ray photoelectron spectroscopy (XPS) with an Al-*K*_*α*_ (1486.8 eV) X-ray source. The specific surface area of the NPs was measured using a nitrogen-adsorption analyser with Brunauer–Emmett–Teller analysis (SSA_BET_).

### Fabrication and Characterization of Gas Sensor

**Fig. 1:**
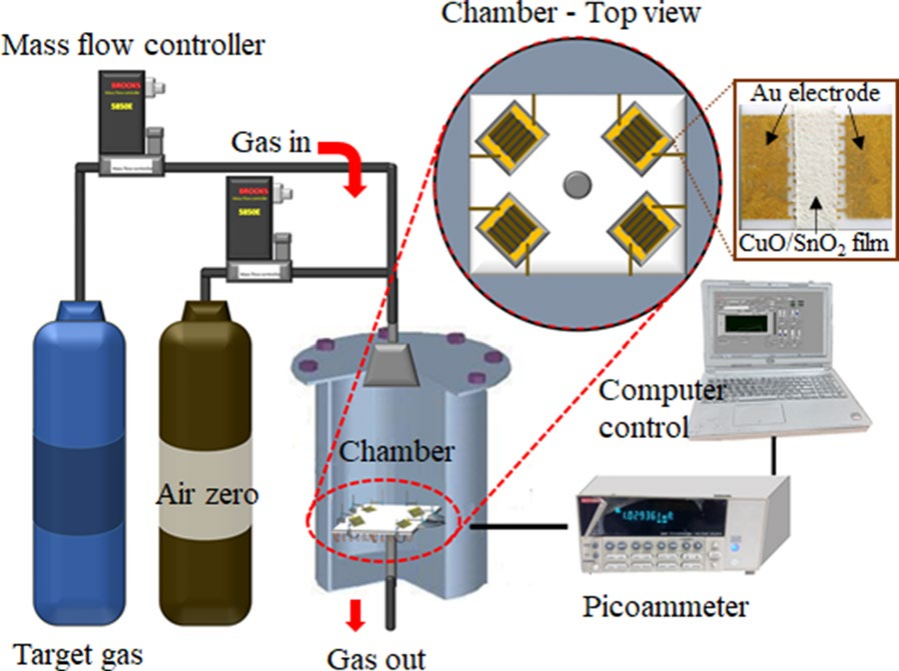
Gas-sensing measurement system with the photograph of a CuO/SnO_2_ sensor

To fabricate SnO_2_ and 5–25 wt% CuO/SnO_2_ sensors, 60 mg of powder was thoroughly mixed with an α-terpineol (Aldrich, 90%)-based vehicle containing ethyl cellulose (30–70 mPa s, Fluka) to produce a homogeneous paste. Next, a sensing film was deposited on an alumina substrate (0.40 × 0.55 × 0.04 cm^3^) with prepatterned interdigitated gold electrodes (0.24 cm × 0.5 cm) by spin coating of the paste at 700 rpm for 10 s and at 3000 rpm for 30 s. The 200-nm-thick interdigitated Au electrodes were deposited on alumina substrates by a sputtering process with argon gas at a pressure of 3 × 10^−3^ mbar. The interdigit spacing, width, and length were 100 μm, 100 μm, and 0.24 cm, respectively. The resulting sensors as illustrated in Fig. 1 were annealed for 2 h at 450 °C at a ramping rate of 4 °C/min to eliminate organic components from the sensing layers. The microstructures of sensing films were characterized using field-emission scanning electron microscopy (FE-SEM) and energy dispersive X-ray analysis (EDS).

### Gas Sensing Measurement

The sensor characteristics toward H_2_S in the concentration range of 0.15–10 ppm was measured in a homemade sealed stainless-steel test chamber with an active volume of 0.5 L (Fig. 1). An external Ni/Cr heater operated with a computer-controlled power supply was used to control the working temperature ranging from 150 to 350 °C. The selectivity properties were evaluated against H_2_, SO_2_, CH_4_ and C_2_H_2_. At a given working temperature, the sensors were initially settled in synthetic dry air for 10 min to obtain a steady resistance in air. Next, the dry air was mixed with a target gas sample to a desired gas concentration at a fixed total flow rate (2 L/min) using computerized multichannel mass flow controllers (Brook Instruments model 5850E). Each gas sample was applied to the sensors for 25 min and the dry air was resumed for 45 min. The sensor resistance was measured by the amperometric method at a bias of 10 V DC using a picoammeter (Kiethley model 6487). The performances of sensors with various Cu concentrations were characterized in terms of gas response, response time, selectivity, and stability. The gas response (*S*) was expressed as *S* = *R*_a_*/R*_g_ for a reducing gas (H_2_S, H_2_, CH_4_, SO_2_ and C_2_H_2_), where *R*_a_ and *R*_g_ were the sensor resistance in air before and after exposure to a target gas, respectively. The response time (*t*_res_) was the time taken to attain 90% of a steady-state response signal upon exposure to a target gas.

## Results and Discussion

### Structural Characteristics of Particles and Sensing Film

**Fig. 2:**
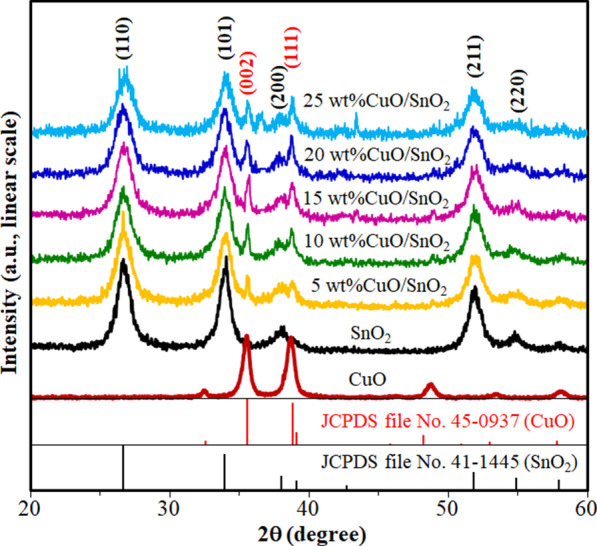
XRD pattern of CuO, SnO_2_ and 5–25 wt% CuO/SnO_2_ NPs

XRD patterns of CuO, SnO_2_, and 5–25 wt% CuO/SnO_2_ NPs are displayed in Fig. 2. The sharp diffraction peaks indicate the crystalline characteristic of all NPs. The diffraction patterns of SnO_2_ and CuO correspond to tetragonal and monoclinic structures according to JCPDS files no 41-1445 and 45-0937, respectively. The SnO_2_ powder exhibits three main peaks, while the CuO powder displays two distinct major peaks. The spectra for the 5–25 wt% CuO/SnO_2_ NPs show the secondary CuO peaks of (002) and (111) planes together with the main SnO_2_ peaks of (111), (101) and (211) planes, demonstrating the coexistence of CuO and SnO_2_ phases. The mean crystallite sizes (*d*) of CuO/SnO_2_ NPs were determined using Scherrer’s equation (*d* = *Kλ*/(*β*cos*θ*) where *K* is the geometric factor of 0.89 for spherical particles, *λ* is the X-ray wavelength and *β* is the full width at half maximum of an XRD peak at the angle, *θ*. The mean crystallite diameter of unloaded SnO_2_ is estimated to be 10 nm, while that of 20 wt% CuO/SnO_2_ NPs is relatively small at 7 nm. The result indicates the inhibition of grain growth due to CuO loading on SnO_2_ NPs. The chemical compositions and oxidation states of CuO and SnO nanoparticles will be evaluated further by EDX and XPS analyses.

**Fig. 3:**
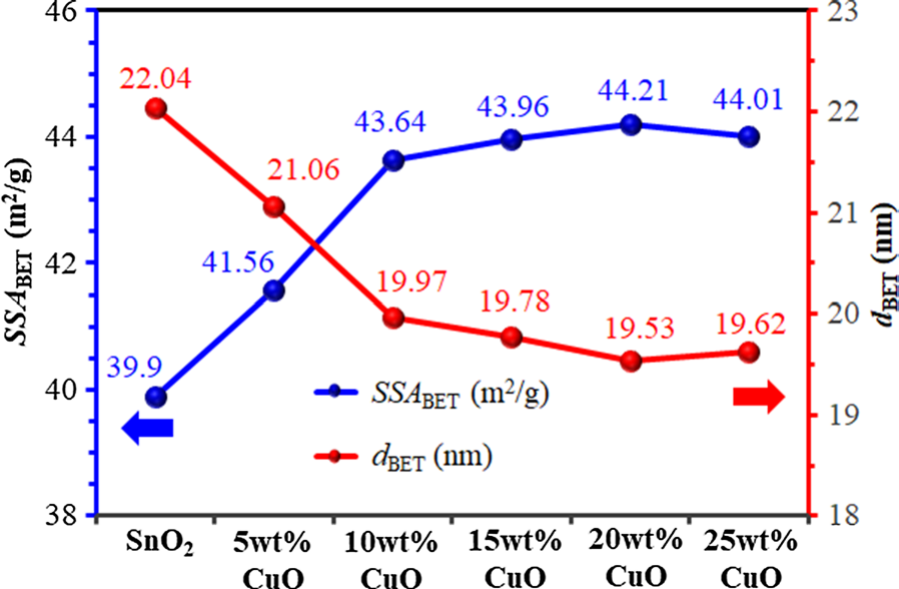
BET specific surface area (SSA_BET_) and particle diameter (*d*_BET_) of SnO_2_ and 5–25 wt% CuO/SnO_2_ NPs

BET specific surface areas (SSA_BET_) and particle diameters (*d*_BET_) of SnO_2_ and 5–25 wt% CuO/SnO_2_ NPs are shown in Fig. 3. SSA_BET_ of CuO/SnO_2_ NPs substantially increases from 39.9 to 44.21 m^2^/g, while the *d*_BET_ reduces accordingly from 22.04 to 19.53 nm as the CuO content rises from 0 to 20 wt%. As the Cu content increases further to 25 wt%, SSA_BET_ decreases slightly to 44.01 m^2^/g and *d*_BET_ increases to 19.62 nm. The results agree with the XRD analysis of crystallite size. The influence of CuO loading level on SSA_BET_ may be attribute to the inclusion of smaller CuO NPs produced by impregnation. The CuO NPs may act as separators to inhibit self-coagulation among SnO_2_ NPs, resulting in the substantial increment of the effective surface area.

**Fig. 4:**
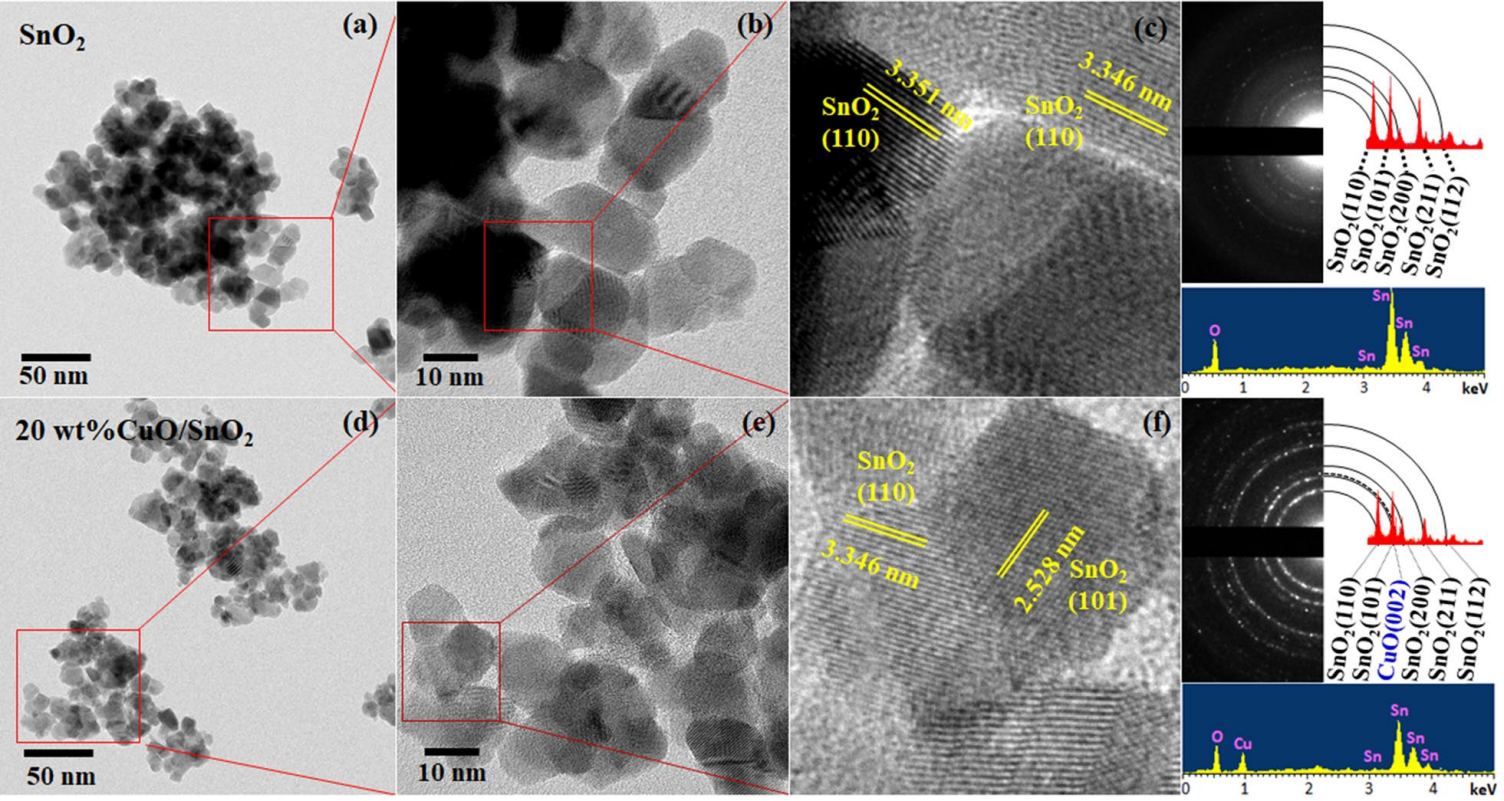
BF-TEM and HR-TEM images with corresponding SAED patterns of **a**–**c** SnO_2_ NPs and **d**–**f** 20 wt% CuO/SnO_2_ NPs

Figure 4 shows typical surface morphologies of coprecipitation/ impregnation synthesized SnO_2_ and 20 wt% CuO/SnO_2_ NPs. The BF-TEM images show that most SnO_2_ particles exhibit spheroidal shapes with different diameters ranging from 5 to 20 nm. After CuO loading, the diameters of SnO_2_ NPs tend to be smaller but the secondary phase of CuO cannot be clearly identified (Fig. 4d–f). The related SAED patterns display dotted ring features of polycrystalline tetragonal SnO_2_ structures with main diffraction rings corresponding to (110), (101), (200), (211) and (112) planes of SnO_2_ as well as (002) plane of CuO in agreement the XRD analysis. The rings related to CuO were quite obscure due likely to weak diffraction signal from very small CuO secondary phase. Correspondingly, the HR-TEM images show lattice fringes on nanoparticles mainly associated with the planes of SnO_2_ crystals. The secondary CuO phase structures cannot be observed in HR-TEM image due possibly to their very small sizes beyond the resolution of the TEM instrument.

**Fig. 5:**
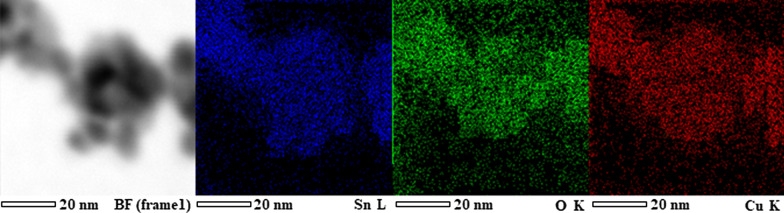
Scanning-TEM (STEM) image and corresponding elemental EDS maps of 20 wt% CuO/SnO_2_ NPs

Scanning transmission electron microscopy (STEM) and high-resolution EDS mapping analysis were employed to investigate the distributions of CuO in 20 wt% CuO/SnO_2_ NPs as presented in Fig. 5. The STEM image illustrates a cluster of roughly round nanoparticles with diameters in the range of 5–15 nm in agreement with the TEM images but with relatively low image resolution due to scanning aberration. The corresponding EDS maps of Sn, O, and Cu elements demonstrate the detailed distribution of these species on various SnO_2_ nanoparticles in the selected area. Apparently, Cu species are widely distributed on particles with similar density to Sn and O species. The results suggest that the CuO secondary nanoparticles are present and closely distributed on SnO_2_ surfaces forming distributed CuO–SnO_2_ junctions within the CuO/SnO_2_ composite. However, the particles and related junctions are very small at molecular scales so that they cannot be exactly discerned by the TEM/STEM characterizations.

**Fig. 6:**
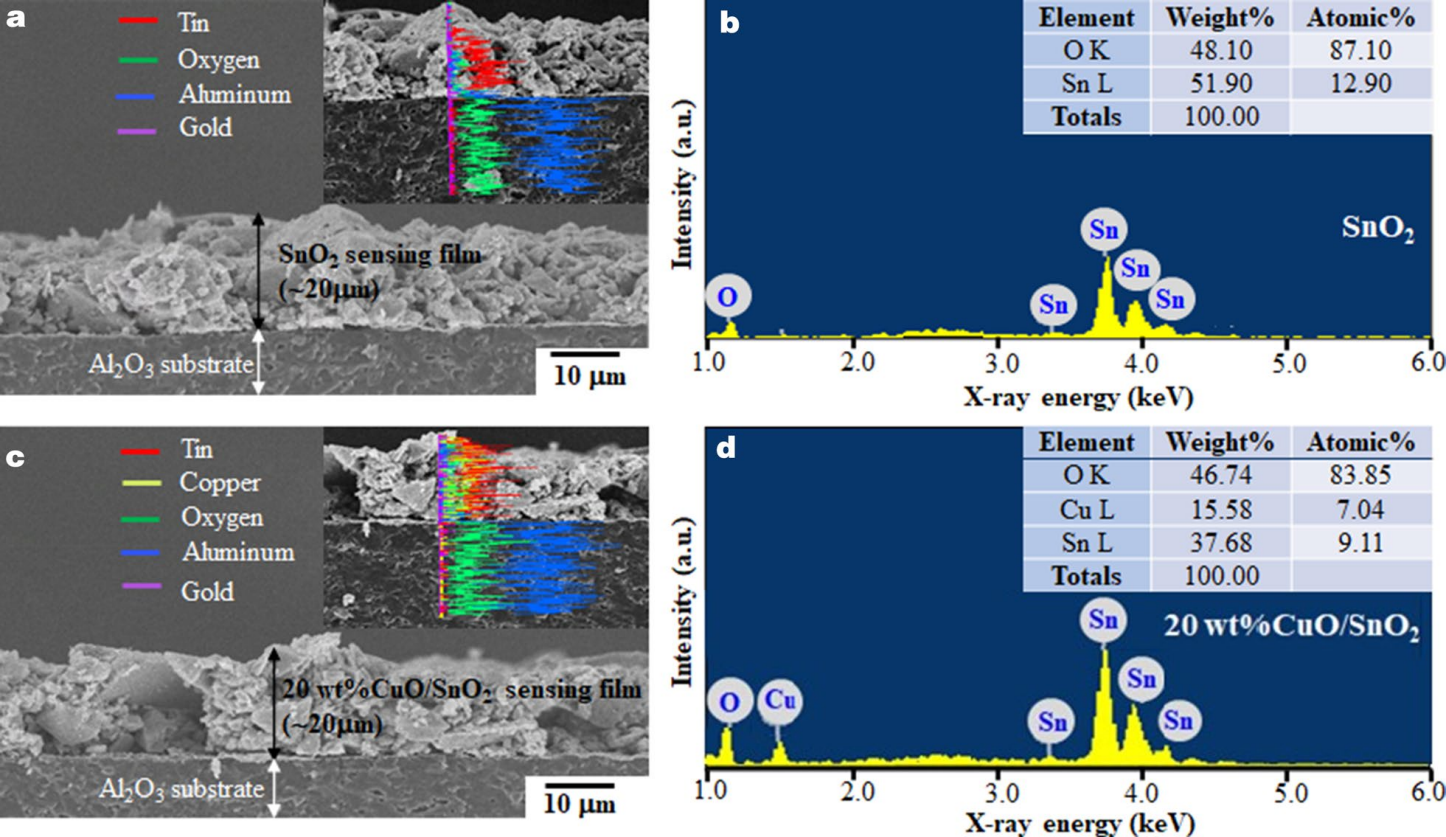
Cross-sectional FE-SEM images with EDS line-scan profiles (inset) and corresponding EDS spectra with elemental composition tables (inset) of the **a**, **b** SnO_2,_ and **c**, **d** 20 wt%CuO/SnO_2_ NPs sensing films

Figure 6 illustrates the cross-sectional morphologies and chemical compositions of SnO_2_ and 20 wt% CuO/SnO_2_ films. Both layers are approximately 20 μm thick and similarly comprise agglomerated particles on solid-textured substrates. The elemental compositions of SnO_2,_ and 20 wt% CuO/SnO_2_ are listed in the inset tables of Fig. 5b, d. It reveals that the atomic contents of Sn and O of SnO_2_ NPs are lower than the expected values (33:67) of stoichiometric SnO_2_. With 20 wt% CuO loading, a Cu peak appears with a high Cu content of ~ 15.6 wt% or 7.04 at%, which is still smaller than that of Sn. Additionally, the Cu content from five different areas is found to vary from 14 to 18 wt%, indicating some variation of chemical composition within the film. Therefore, CuO loading by impregnation does not markedly influence particle morphologies but considerably changes the elemental composition.

**Fig. 7:**
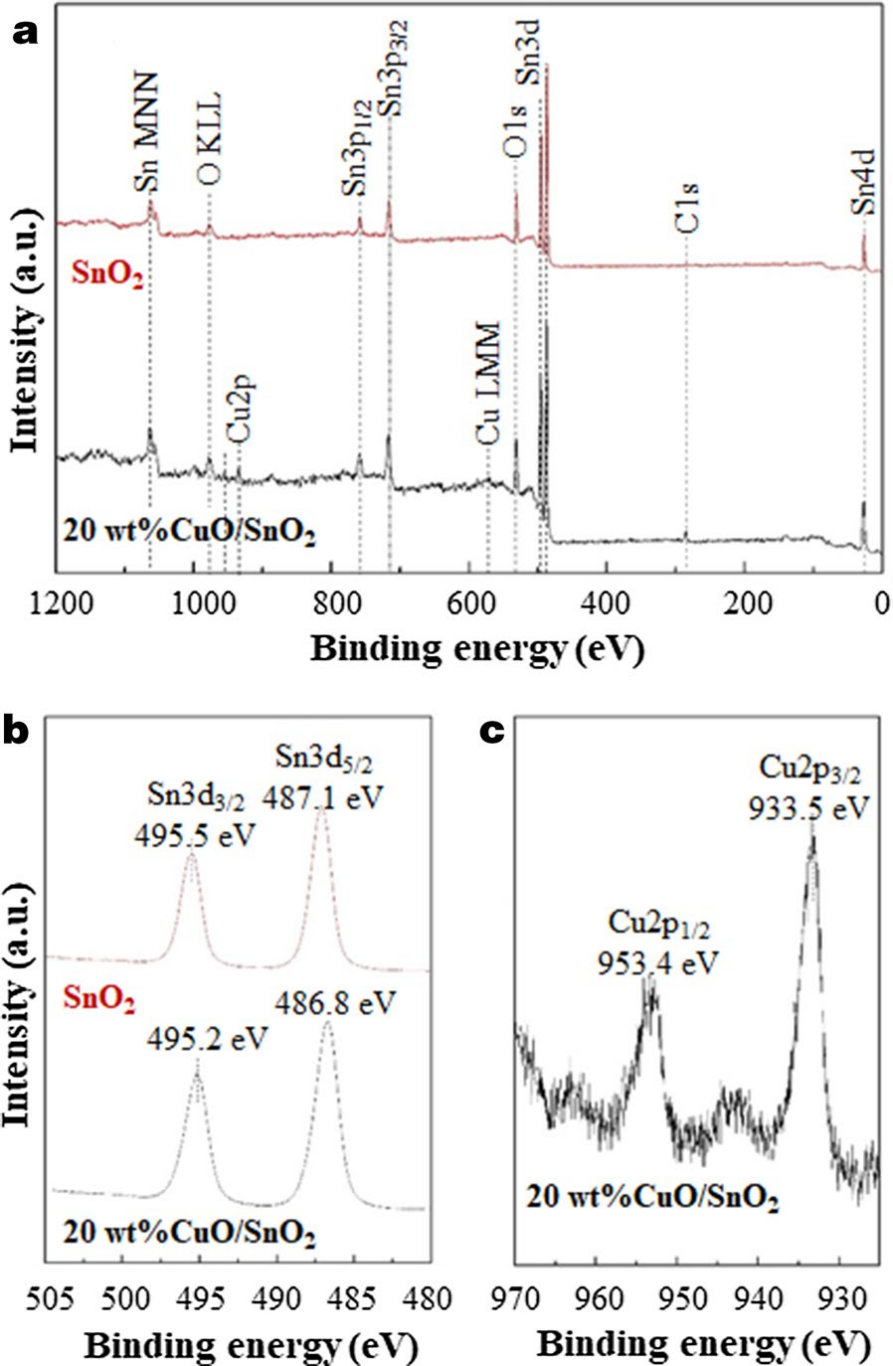
**a** Survey and high-resolution XPS spectra of the **b** Sn3d and **c** Cu 2*p* core levels of SnO_2_ and 20 wt% CuO/SnO_2_ NPs

Figure 7 presents the oxidation states of elements in SnO_2_ and 20 wt% CuO/SnO_2_ NPs. The XPS survey spectrum of SnO_2_ reveals the presence of C, O and Sn, while that of 20 wt% CuO/SnO_2_ demonstrates the existence of C, O, Sn and Cu. The results confirm the formation of CuO/SnO_2_ composites with typical organic/carbon contaminations on surfaces. For Sn element, the Sn3d_5/2_ and Sn3d_3/2_ doublet peaks of SnO_2_ and 20 wt% CuO/SnO_2_ NPs are similarly observed at the binding energies of 486.8–487.1 eV and 495.2–495.5 eV, respectively. The peak locations can be assigned to the Sn^4+^ oxidation state of SnO_2_ [29]. In the case of 20 wt% CuO/SnO_2_ NPs, the Cu2p core levels comprise Cu2p^3/2^ and Cu2p^1/2^ peaks centred at 933.5 eV and 953.4 eV along with the satellite peaks at ~ 942.9 and ~ 964.2 eV, corresponding to the Cu^2+^ oxidation state of CuO [30]. The observed oxidation states affirm the coexistence of CuO and SnO_2_ structures.

### Gas-Sensing Characteristics

**Fig. 8:**
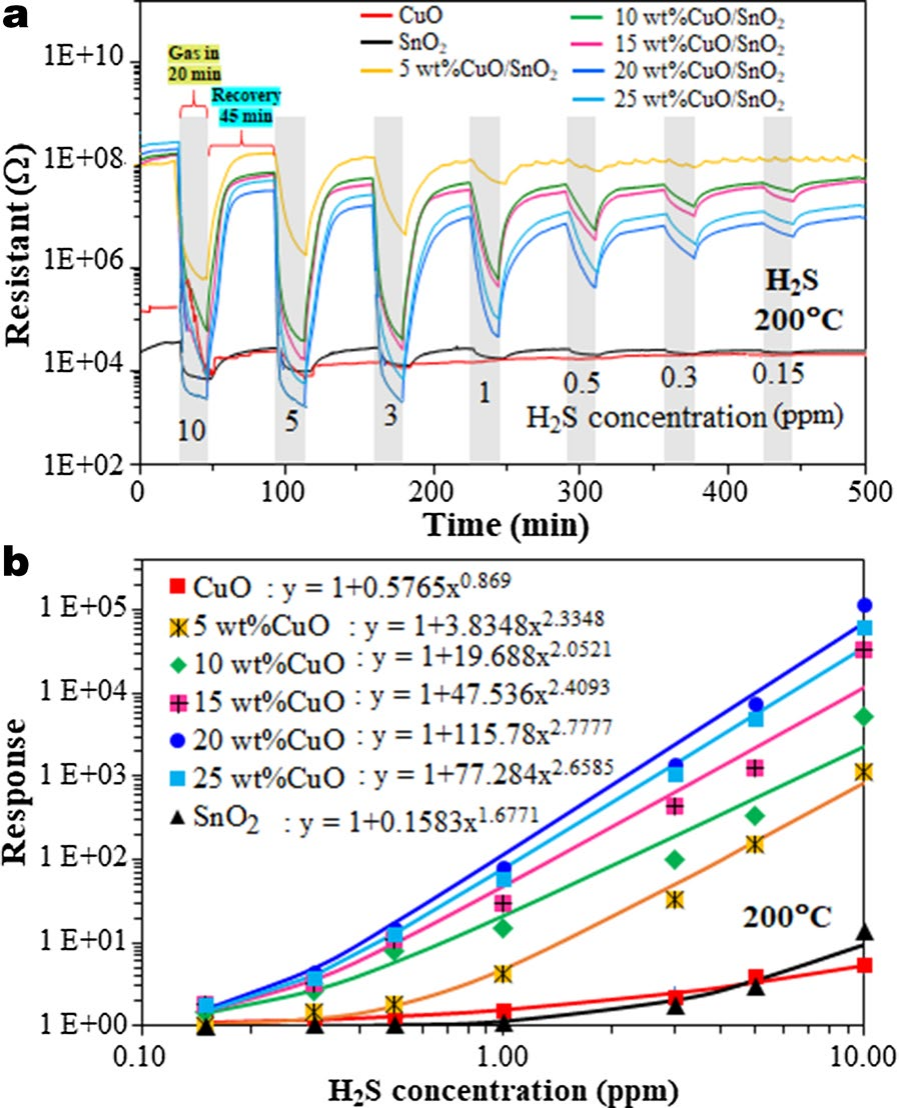
**a** Dynamic response of CuO, SnO_2_ and 5–25 wt%CuO/SnO_2_ gas sensors subjected to 0.15 to 10 ppm H_2_S pulses at 200 °C and **b** corresponding sensor response vs. H_2_S concentration

Figure 8a displays the changes in resistance of CuO, SnO_2_ and 5–25 wt% CuO/SnO_2_ films subjected to H_2_S pulses with varying concentrations from 0.15 to 10 ppm at a working temperature of 200 °C. The resistance in air of SnO_2_ film increases by more than two orders of magnitude after loading CuO with 5–25 wt% contents. Additionally, it is observed that the baseline resistances of various CuO/SnO_2_ sensors are not very different and only tend to increase slightly with increasing CuO loading level. To identify whether the resistance is changed due to the film geometry or material related properties, the film resistivity was additionally measured by the well-known four-probe method using 4-stripe Au/Cr electrodes with an interelectrode spacing of 100 μm and a bias current of 0.1 μA. The measured average resistivity values of CuO, SnO_2_, and 5–25 wt% CuO/SnO_2_ films in air at 350 °C are ~ 8.1 × 10^3^, 2.1 × 10^4^ and 7.4 × 10^7^ − 1.8 × 10^8^ Ω cm, respectively. The results confirm significant differences in resistivity among the three sets of materials and the similarities in resistivity among 5–25 wt% CuO/SnO_2_ films. This behavior may be explained based on two effects including the percolation breaking of aggregated SnO_2_ nanoparticles due to CuO secondary nanoparticles and the formation of CuO/SnO_2_ (p–n) heterojunctions. The TEM/HR-TEM/STEM data suggest that CuO secondary nanoparticles may be formed surrounding the SnO_2_ nanoparticles, thus breaking the percolation of agglomerated SnO_2_ particles and forcing most conduction paths to be across the CuO nanoparticles. In addition, the formation of CuO/SnO_2_ heterojunctions may induce carrier depletion regions throughout secondary CuO nanoparticles due to the work function difference, creating highly resistive conduction paths. Thus, an addition of CuO to SnO_2_ particles at the level above the minimum value required to break the percolation of SnO_2_ particles will cause a large increase of resistance as fully depleted CuO particles block electrical conduction. The lowest Cu content in this study of 5% is quite substantial and thus likely to exceed the percolation breaking threshold. Further addition of CuO will only slightly increase the resistance since the electrical conduction via fully depleted CuO is already nearly minimal. Other effects including particle/grain sizes, film thickness, electrode separation, and electrode contact may be neglected since they are not greatly changed according to the structural characterization results. Upon exposure to H_2_S, the sensor resistances decrease rapidly before recovering to the baseline levels after the resumption of dry air, confirming a typical n-type sensing characteristic. Interestingly, the baseline resistance of CuO sensor considerably drifts downward after several H_2_S pulses in contrast to the SnO_2_ sensor that shows insignificant baseline drift. In the case of CuO/SnO_2_ sensors, the baseline drift tends to increase with increasing Cu content. These behaviors may be related to the slow and incomplete CuO–CuS transformative reactions to be further discussed in Sect. 3.3.

The corresponding sensor response plotted versus H_2_S concentration at 200 °C is shown in Fig. 8b. All sensor responses increase monotonically with increasing H_2_S concentration. The response characteristics of all sensors conform well to the power law according to the equations as displayed along with the inset labels in Fig. 8b. The power-law exponent of CuO is close to 1, while that of SnO_2_ sensors is around 1.5 and those of CuO-loaded SnO_2_ sensors are larger than 2, suggesting differences in H_2_S reaction mechanisms on the surfaces of these materials [31]. Furthermore, the sensor response increases greatly as the CuO content increases from 0 to 20 wt% before slightly declining at a higher CuO content of 25 wt% and the 20 wt% CuO/SnO_2_ sensor offers the highest response of 1.36 × 10^5^ to 10 ppm H_2_S at 200 °C. Moreover, it exhibits decent responses of ~ 2, 5, 20 and 230 at the lower H_2_S concentrations of 0.15, 0.3, 0.5 and 1 ppm, respectively. The excellent performances of 20 wt% CuO/SnO_2_ sensor may be attributed to the increase of specific surface area due to CuO loading and the formation of CuO/SnO_2_ heterojunctions to be further discussed in the next section.

**Fig. 9:**
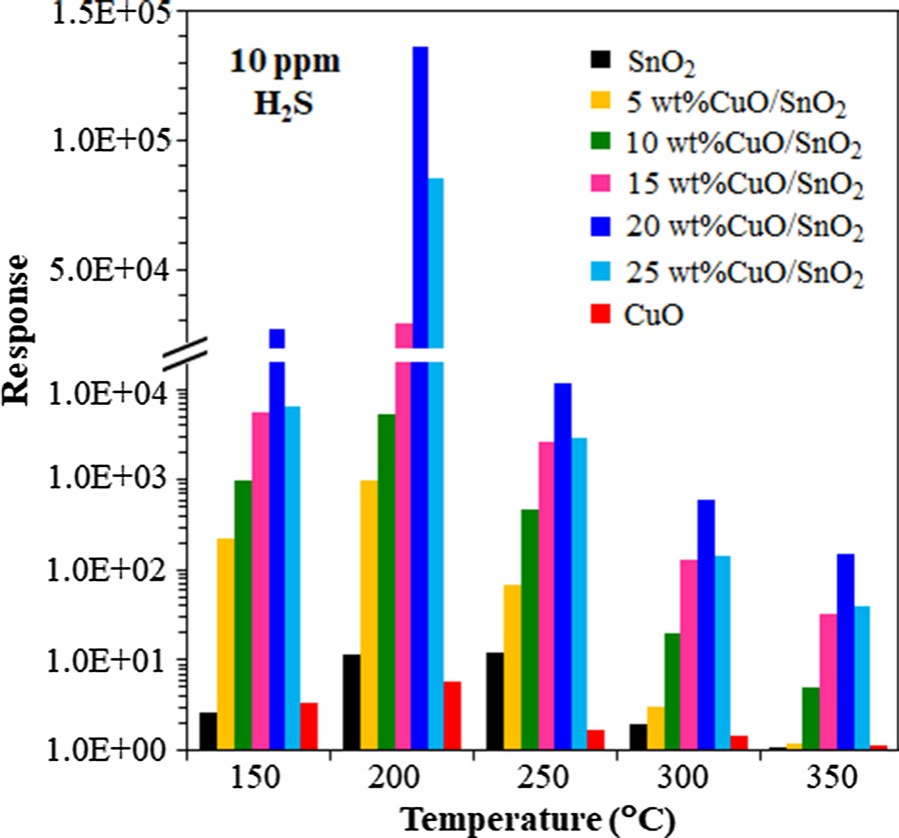
Effect of working temperature on response to 10 ppm H_2_S of CuO, SnO_2_ and 5–25 wt%CuO/SnO_2_ sensors

Figure 9 presents the plot of the response versus working temperature of unloaded and CuO-loaded SnO_2_ sensors at a H_2_S concentration of 10 ppm. The H_2_S responses of CuO/SnO_2_ NPs sensors increase significantly with the increasing temperature from 150 to 200 °C and then reduce rapidly when the temperature further rises. Hence, 200 °C is the optimal working temperature of the CuO-loaded SnO_2_ sensors. Specifically, the optimal 20 wt% CuO/SnO_2_ sensor gives the highest response of 1.36 × 10^5^, which is much higher than those of other sensors at 200 °C. The optimal working temperature of 200 °C corresponds to the temperature that maximizes the H_2_S adsorption rate relative to desorption rate of CuO/SnO_2_ surfaces. Furthermore, 5–25 wt% CuO/SnO_2_ sensors display a lower optimal working temperature than that of SnO_2_ sensor (250 °C). The relatively low optimal working temperature will be subsequently explicated by CuO loading effects.

**Fig. 10:**
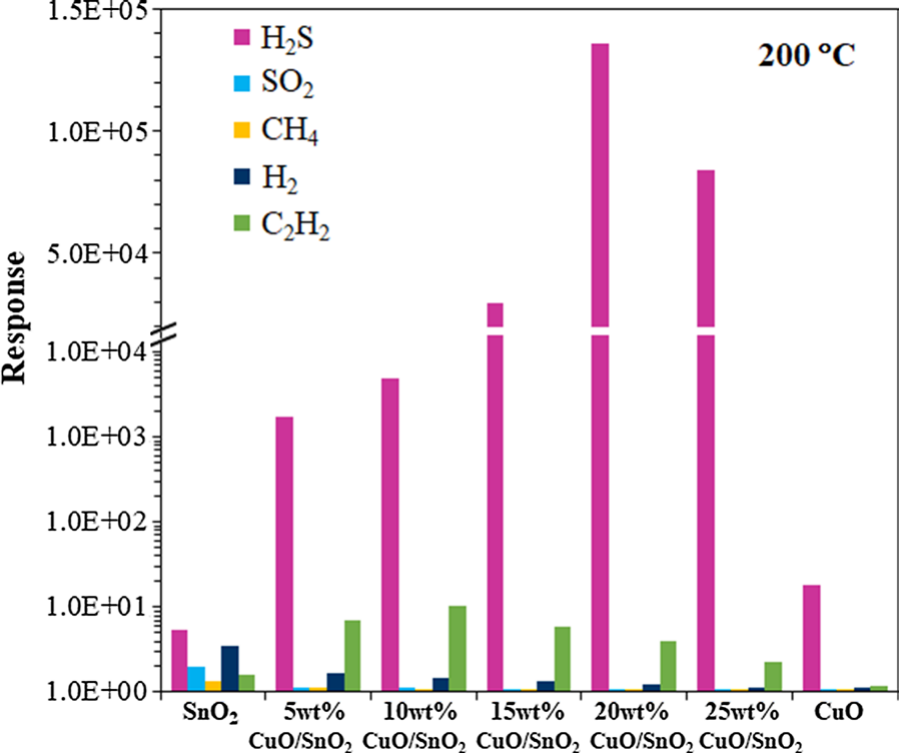
Responses of CuO, SnO_2_ and 5–25 wt%CuO/SnO_2_ sensors toward H_2_S (10 ppm), SO_2_ (200 ppm), CH_4_ (1000 ppm), H_2_ (1000 ppm) and C_2_H_2_ (1000 ppm) at 200 °C

Figure 10 summarizes the H_2_S selectivity of 0–25 wt% CuO/SnO_2_ sensors against SO_2_, H_2_, CH_4_ and C_2_H_2_. This type of sensor exhibits the highest H_2_S selectivity, i.e., more than three orders of magnitude higher H_2_S response than those of other gases. The data prove that CuO is the catalyst that selectively accelerates the reaction with H_2_S. The selectivity behavior may also be attributed to the increase of active sites for H_2_S adsorption due to the highest specific surface area of 20 wt% CuO/SnO_2_ NPs. The enhancements for other tested gases are not significant due probably to relatively weak interactions between gas molecules and 20 wt% CuO/SnO_2_ NPs. The attained H_2_S responses of 20 wt% CuO/SnO_2_ sensors are substantially better than those of many other metal-loaded SnO_2_ and CuO-loaded SnO_2_ sensors made by distinct techniques as listed in Table 1. However, the achieved optimal working temperature of 200 °C is higher than the values of some reports at 100–150 °C. The lower working temperature is generally preferred in practical applications. Nevertheless, the 20 wt% CuO/SnO_2_ sensor may operate at a lower working temperature of 150 °C where the sensor still exhibits a high response of 3.1 × 10^4^ to 10 ppm H_2_S (Fig. 9), which is also higher than the response values of other sensors reported in Table 1. Therefore, the CuO-loaded SnO_2_ sensor is a highly promising candidate for H_2_S sensing due to its high H_2_S response, high H_2_S selectivity and low working temperature.

Finally, the stability, repeatability, and reproducibility of CuO/SnO_2_ sensors were evaluated from four samples produced in the same batch. All sensors exhibited good stability with less than 15% drift in sensor response over 1 month under the same operating conditions. Moreover, each sensor showed good repeatability with less than 12% response variation from 8 repeated measurements. In addition, four sensors from the same batch were found to have fair response variation of less than 26% evaluated under the same test condition.

### Gas-Sensing Mechanisms

**Fig. 11:**
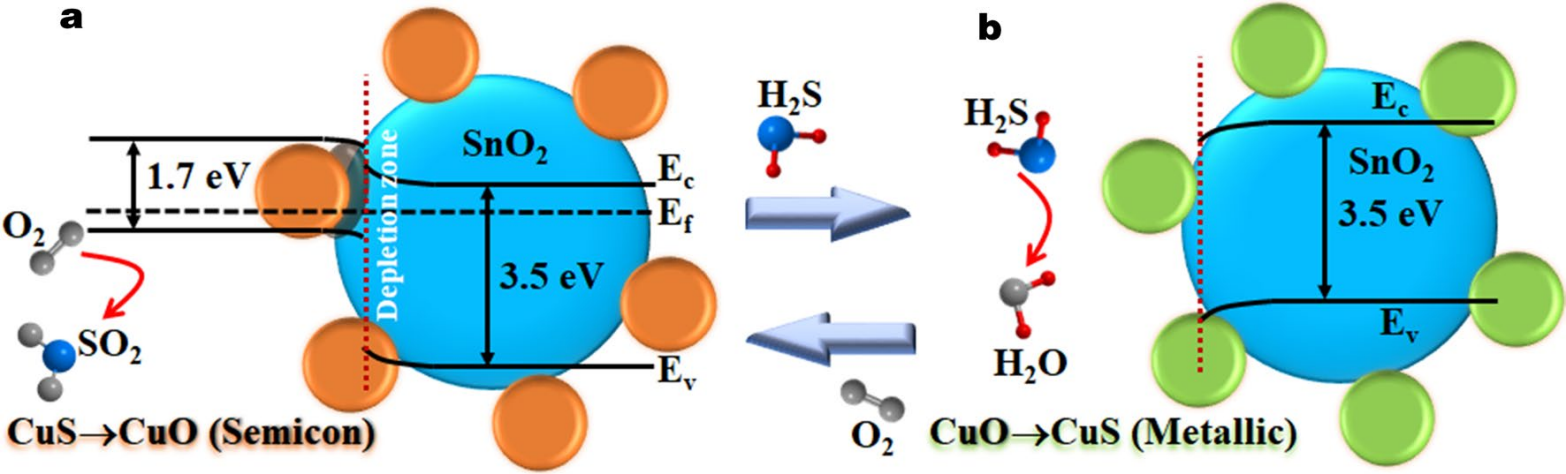
Energy band diagrams of CuO/SnO_2_ heterojunctions **a** before and **b** after exposure to H_2_S (*E*_f_ = Fermi-energy level, *E*_C_ = conduction band and *E*_V_ = valence band)

The characterization results suggest the formation of CuO/SnO_2_ composite comprising very small CuO species on SnO_2_ nanoparticles. Thus, the mechanisms for electrical response of CuO/SnO_2_ sensing films toward H_2_S may be described based on the composite junction theory of p-n junctions at the contacts between p-type CuO and n-type SnO_2_ as depicted in Fig. 11. For undoped SnO_2_, chemisorbed oxygen species (O^−^) are formed resulting in the creation of depletion regions on surface at a moderate temperature. Upon exposure to H_2_S, H_2_S molecules interact with adsorbed oxygen species on SnO_2_ surface (H_2_S + 3O^−^ → H_2_O + SO_2_ + e^−^), releasing electrons to SnO_2_ conduction band and reducing the sensor resistance. At a low working temperature of 200 °C, the concentration of oxygen species is very low, leading to a low reaction rate and a low H_2_S response. With CuO loading, additional depletion regions will be formed at various p-n junctions around the surface of SnO_2_ nanoparticles. In addition, carriers in secondary CuO nanoparticles, which can break percolation of aggregated SnO_2_ nanoparticles, may be fully depleted, resulting in a high electrical resistance in air. In ambient with H_2_S, the gas molecules can additionally react with the catalytic CuO NPs, leading to the creation of copper sulfide (CuS) via the reaction (Eq. 1) [26]:

1 $${\text{CuO}} + {\text{H}}_{2} {\text{S}} \to {\text{CuS}} + {\text{H}}_{2} {\text{O}}$$

CuS is more conductive than CuO, leading to lower potential barriers at depletion regions around the interfaces. The induction of metallic CuS is equivalent to the injection of free electrons into the p-type material (CuO), making it less p-type. This encourages the electron transfer from CuS to SnO_2_, resulting in additional decrease of depletion width and increase of the electrical conductance of SnO_2_. The decrease of resistance due to the formation of CuS is much larger than the reduction due to the reducing reaction with oxygen species due to transfer of more electrons from CuS. At low CuO contents, there are relatively few and small CuO nanoparticles that are fully transformed into CuS surrounding SnO_2_ particles. It will provide a limited amount of electrons to SnO_2_ due to relatively few heterojunctions, resulting in small reduction of depletion region widths in SnO_2_ and small resistance drop upon H_2_S exposure. As the CuO content increases, the numbers of transformed CuS nanoparticles and heterojunctions increase, leading to an increased number of conduction paths through CuS as well as much reduced SnO_2_ depletion region widths and thus a higher resistance drop that can be achieved after H_2_S exposure. However, CuO particles may coalesce into large ones and the number of CuO/SnO_2_ heterojunctions becomes lower at very high CuO content (> 20 wt%). The large CuO particles will not be fully transformed to CuS due to limited reaction depth with H_2_S and the depletion regions in CuO cores remain, limiting the conduction through CuO and reducing attainable resistance drop. In the case of CuO, the response is low despite the formation of CuS because the resistance of CuO is already low and is not much higher than that of CuS [20]. After H_2_S in atmosphere extinguishes, the electrical resistance returns to its original values as CuS can be reoxidized to CuO in air at an elevated temperature according to the reaction (Eq. 2) [26]:

2 $${\text{CuS}} + {\text{O}}_{2} \to {\text{CuO}} + {\text{SO}}_{2}$$

The oxidation of CuS is slow at a low working temperature. As the increase of working temperature, the oxidation rate increases and lead to the increase of recovery rate. Since the CuS–CuO transformative reaction (Eq. (2)) is slower than the CuO–CuS one (Eq. (1)) at this working temperature, residual CuS materials can remain after subjecting CuO to several H_2_S pulses. This results in a substantial downward baseline drift of CuO sensor and the increase of baseline drift with increasing Cu content of CuO/SnO_2_ sensors as previously observed in Fig. 8a. However, there is an exception in the case of 5 wt% CuO/SnO_2_ sensor, which exhibits a small upward drift of baseline resistance after the first pulse. It may occur because the sensor did not fully reach the steady state before applying the first pulse leading to some upward recovery owing to oxidation in air while the drift due to CuS–CuO transformation at this low Cu content is relatively small due to a low residual CuS content. At higher Cu contents, the downward drifts due to residual CuS are large and overwhelm the small upward recovery. The baseline drift considerably reduces the validity, repeatability and stability of sensor response of CuO/SnO_2_ as the response to a subsequent H_2_S pulse is affected by the residual CuS concentration after the previous H_2_S exposure leading to negative deviations from the ideal response behavior. Thus, the calculated responses of the CuO/SnO_2_ sensors especially with high Cu contents in Fig. 8b are lower the theoretical values under no residual CuS condition. The problems can be reduced by increasing the working temperature. Thus, the sensors may operate above the optimal working temperature at 250 °C when the drift is low, and response is still high. CuS structure can be formed at 103 °C and will be transformed into Cu_2_S, a less conductive ionic conductor, at the temperature above 220 °C [26]. Consequently, the sensor response of CuO/SnO_2_ NPs decreases when the temperature rises above 200 °C. The observed high H_2_S selectivity against SO_2_, H_2_, CH_4_ and C_2_H_2_ can also be explained in relation to the working temperature. At the optimal working temperature of 200 °C, the rate of CuO–CuS transformation is high, while the reducing reaction rates of SO_2_, H_2_, CH_4_ and C_2_H_2_ are very low because these reactions require the chemisorbed oxygen species whose density is still very low at this working temperature.

## Conclusions

0–25 wt% CuO/SnO_2_ NPs were fabricated using the precipitation and impregnation method. XRD, BET, TEM, SEM, EDS and XPS data suggested the loading of very small CuO nanoparticles on larger SnO_2_ NPs. The gas-sensing results demonstrated that CuO loading greatly enhanced the H_2_S response of SnO_2_ NPs with an optimal Cu content of 20 wt%. The 20 wt%CuO/SnO_2_ sensor can perceive low-ppm H_2_S concentrations with ultra-high responses (1.4 × 10^5^ at 10 ppm), short response times (35 s), fair recovery times (a few minutes), very high H_2_S selectivity against SO_2_, CH_4_, H_2_ and C_2_H_2_ and good stability. They could also offer a wide detection range (0.15–10 ppm) when compared with the unloaded one (3–10 ppm). Therefore, the CuO/SnO_2_ sensors synthesized by precipitation and impregnation could be a promising candidate for H_2_S detection in environmental applications.

## Data Availability

The datasets supporting the conclusions of this article are included in the article.

## Abbreviations

NPs: Nanoparticles
XRD: X-ray diffraction
HR-TEM: High-resolution transmission electron microscopy
FE-SEM: Field-emission scanning electron microscopy
EDX: Energy-dispersive X-ray spectroscopy
AFM: Atomic force microscopy
BET: Brunauer–Emmett–Teller
SSA_BET_: Specific surface area
XPS: X-ray photoelectron spectroscopy
